# Tris(allyl­thio­urea-κ*S*)bromidozinc(II) bromide

**DOI:** 10.1107/S1600536811011809

**Published:** 2011-04-07

**Authors:** Hai-Qing Sun, Xin-Qiang Wang, Tao Jin

**Affiliations:** a School of Materials Science and Engineering, Shandong University of Science and Technology, Qingdao 266510, People’s Republic of China; b State Key Laboratory of Crystal Materials, (Shandong University), Jinan 250100, People’s Republic of China

## Abstract

In the title compound, [ZnBr(C_4_H_8_N_2_S)_3_]Br, the Zn^II^ atom is coordinated by one Br atom and the S atoms of three *N*-allyl­thio­urea ligands in a distorted tetra­hedral geometry. The Zn^II^ atom and the two Br atoms are located on a threefold axis.

## Related literature

For transition metal complexes containing allyl­thio­urea ligands, see: Gambino *et al.* (2002 ▶); Olijnyk *et al.* (2003 ▶). For similar structures of *N*-allyl­thio­urea coordination compounds, see: Zhang *et al.* (1990 ▶); Yuan *et al.* (1990 ▶); Hou *et al.* (1993 ▶); Sun *et al.* (2004 ▶). For compounds that have similar Zn—Br bond lengths, see: Bermejo *et al.* (2000 ▶, 2001 ▶); Castineiras *et al.* (2000 ▶). 
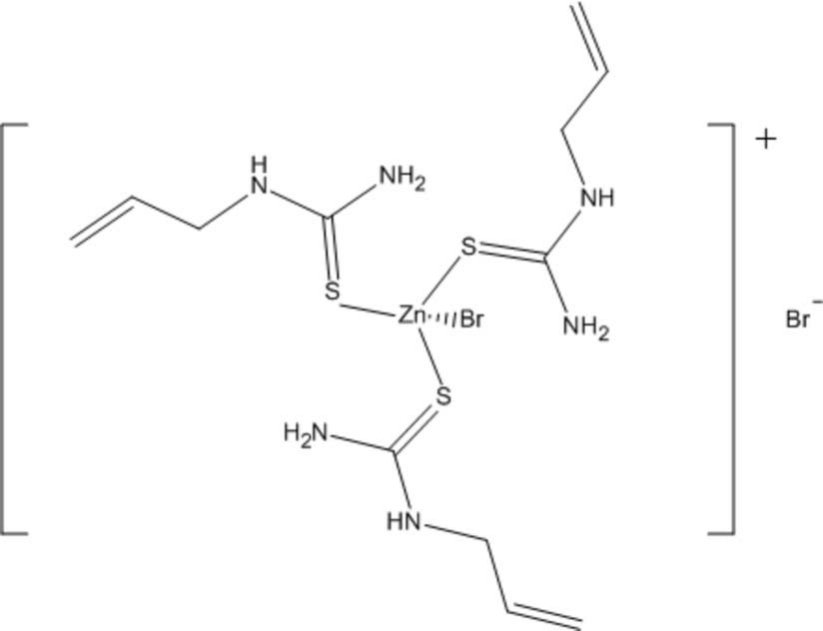


## Experimental

### 

#### Crystal data

[ZnBr(C_4_H_8_N_2_S)_3_]Br
*M* *_r_* = 573.74
Trigonal, 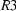

*a* = 11.3591 (2) Å
*c* = 14.5172 (4) Å
*V* = 1622.19 (6) Å^3^
*Z* = 3
Mo *K*α radiation
μ = 5.13 mm^−1^
*T* = 296 K
0.35 × 0.32 × 0.32 mm

#### Data collection

Bruker APEXII CCD area-detector diffractometer
Absorption correction: multi-scan (*SADABS*; Bruker, 2005 ▶) *T*_min_ = 0.265, *T*_max_ = 0.294
2605 measured reflections
1359 independent reflections
1305 reflections with *I* > 2σ(*I*)
*R*_int_ = 0.018

#### Refinement

*R*[*F* ^2^ > 2σ(*F* ^2^)] = 0.019
*wR*(*F* ^2^) = 0.045
*S* = 0.95
1359 reflections
73 parameters
1 restraint
H-atom parameters constrained
Δρ_max_ = 0.25 e Å^−3^
Δρ_min_ = −0.33 e Å^−3^
Absolute structure: Flack (1983 ▶), 522 Friedel pairs
Flack parameter: 0.047 (8)

### 

Data collection: *APEX2* (Bruker, 2005 ▶); cell refinement: *SAINT* (Bruker, 2005 ▶); data reduction: *SAINT*; program(s) used to solve structure: *SIR97* (Altomare *et al.*, 1999 ▶); program(s) used to refine structure: *SHELXL97* (Sheldrick, 2008 ▶); molecular graphics: *SHELXTL*; software used to prepare material for publication: *WinGX* (Farrugia, 1999 ▶).

## Supplementary Material

Crystal structure: contains datablocks I, global. DOI: 10.1107/S1600536811011809/rn2081sup1.cif

Structure factors: contains datablocks I. DOI: 10.1107/S1600536811011809/rn2081Isup2.hkl

Additional supplementary materials:  crystallographic information; 3D view; checkCIF report

## Figures and Tables

**Fig. 1.**
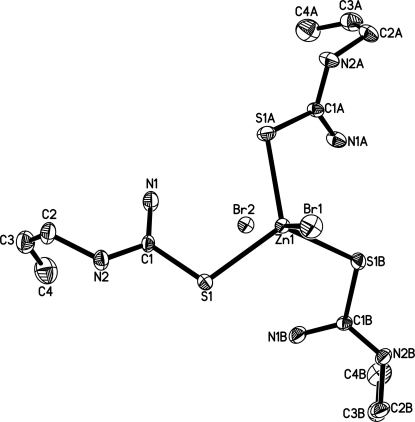
The molecule structure of ATZB with 30% displacement ellipsoids. H atoms are omitted for clarity. [Symmetry codes: (A)-y + 1, x-y, z; (B)-x + y+1, -x + 1, z]

## supplementary crystallographic information

### Comment

Coordination compounds with *N*-allylthiourea (abbreviated as ATU) ligands have many kinds of applications, such as electroplating (Gambino *et al.*, 2002), radiotherapeutic (Olijnyk *et al.*, 2003) and nonlinear optical materials(Zhang *et al.*, 1990; Yuan *et al.*, 1990; Hou *et al.*, 1993; Sun *et al.*, 2004).

The title compound consists of [ZnBr(ATU)_3_]^+^ and Br^-^ ions. The Zn^II^ atom is coordinated to a Br atom and three ATU ligands through their S atoms in a distorted tetrahedral arrangement. The bond angles around the Zn atom range from 101.64 (2)° to 116.038 (14)°, which show an obvious deviation from the ideal tetrahedral value of 109.5°. Zn and Br1 atoms lie on the threefold axis which is perpendicular to the plane of the three S atoms. The Br2 atom also lies on the axis(Fig.1). The Zn—Br1 distance [2.4640 (6) Å] is comparable with the average values reported in other complexes containing Zn—Br bonds, *e.g.* 2.4367 (9) and 2.445 (1)Å (Bermejo *et al.*, 2001), 2.4394 (8) and 2.4457 (7)Å (Castineiras *et al.*, 2000), 2.4207 (7) and 2.4654 (8)Å (Bermejo *et al.*, 2000).

### Experimental

To 2.252 g ZnBr_2_ (0.01 mol) in 5 ml water, 3.486 g ATU (0.03 mol) in 10 ml water was slowly added with stirring. After standing for 1 h, the lower layer of oily solid was separated and dissolved in small volume of ethanol. Small single crystals of Zn[Br(ATU)_3_]Br were obtained by slow evaporation of this solution.

### Refinement

H atoms were placed geometrically (C—H = 0.93 - 0.97 Å, N—H = 0.86 Å) and refined using the riding model approximation, with *U*_iso_ = 1.2*U*_eq_.

### Crystal data

**Table:**

[ZnBr(C_4_H_8_N_2_S)_3_]Br	*D*_x_ = 1.762 Mg m^−^^3^
*M**_r_* = 573.74	Mo *K*α radiation, λ = 0.71073 Å
Trigonal, *R*3	Cell parameters from 2071 reflections
*a* = 11.3591 (2) Å	θ = 2.5–27.5°
*c* = 14.5172 (4) Å	µ = 5.13 mm^−^^1^
*V* = 1622.19 (6) Å^3^	*T* = 296 K
*Z* = 3	Block, colourless
*F*(000) = 858	0.35 × 0.32 × 0.32 mm

### Data collection

**Table:**

Bruker APEXII CCD area-detector diffractometer	1359 independent reflections
Radiation source: fine-focus sealed tube	1305 reflections with *I* > 2σ(*I*)
graphite	*R*_int_ = 0.018
φ and ω scans	θ_max_ = 27.5°, θ_min_ = 2.5°
Absorption correction: multi-scan (*SADABS*; Bruker, 2005)	*h* = −13→14
*T*_min_ = 0.265, *T*_max_ = 0.294	*k* = −14→10
2605 measured reflections	*l* = −15→18

### Refinement

**Table:**

Refinement on *F*^2^	Secondary atom site location: difference Fourier map
Least-squares matrix: full	Hydrogen site location: inferred from neighbouring sites
*R*[*F*^2^ > 2σ(*F*^2^)] = 0.019	H-atom parameters constrained
*wR*(*F*^2^) = 0.045	*w* = 1/[σ^2^(*F*_o_^2^) + (0.*P*)^2^] where *P* = (*F*_o_^2^ + 2*F*_c_^2^)/3
*S* = 0.95	(Δ/σ)_max_ = 0.001
1359 reflections	Δρ_max_ = 0.25 e Å^−^^3^
73 parameters	Δρ_min_ = −0.33 e Å^−^^3^
1 restraint	Absolute structure: Flack (1983), 522 Friedel pairs
Primary atom site location: structure-invariant direct methods	Flack parameter: 0.047 (8)

### Special details

**Table:**

Geometry. All e.s.d.'s (except the e.s.d. in the dihedral angle between two l.s. planes) are estimated using the full covariance matrix. The cell e.s.d.'s are taken into account individually in the estimation of e.s.d.'s in distances, angles and torsion angles; correlations between e.s.d.'s in cell parameters are only used when they are defined by crystal symmetry. An approximate (isotropic) treatment of cell e.s.d.'s is used for estimating e.s.d.'s involving l.s. planes.
Refinement. Refinement of *F*^2^ against ALL reflections. The weighted *R*-factor *wR* and goodness of fit *S* are based on *F*^2^, conventional *R*-factors *R* are based on *F*, with *F* set to zero for negative *F*^2^. The threshold expression of *F*^2^ > σ(*F*^2^) is used only for calculating *R*-factors(gt) *etc*. and is not relevant to the choice of reflections for refinement. *R*-factors based on *F*^2^ are statistically about twice as large as those based on *F*, and *R*- factors based on ALL data will be even larger.

### Fractional atomic coordinates and isotropic or equivalent isotropic displacement parameters (Å^2^)

**Table:**

	*x*	*y*	*z*	*U*_iso_*/*U*_eq_	
Zn1	0.3333	0.6667	0.59387 (3)	0.02676 (12)	
Br2	0.3333	0.6667	0.33457 (3)	0.03334 (12)	
Br1	0.3333	0.6667	0.76360 (3)	0.04428 (15)	
S1	0.19315 (8)	0.43514 (7)	0.56132 (5)	0.03569 (18)	
N2	0.2489 (3)	0.2409 (2)	0.53827 (17)	0.0356 (5)	
H2	0.1781	0.1993	0.5727	0.043*	
N1	0.3941 (3)	0.4382 (2)	0.46202 (16)	0.0380 (6)	
H1A	0.4379	0.4009	0.4402	0.046*	
H1B	0.4190	0.5211	0.4483	0.046*	
C2	0.3134 (3)	0.1641 (3)	0.5097 (2)	0.0397 (7)	
H2A	0.2822	0.0861	0.5501	0.048*	
H2B	0.4107	0.2207	0.5188	0.048*	
C1	0.2880 (3)	0.3680 (3)	0.51681 (16)	0.0280 (5)	
C3	0.2890 (4)	0.1143 (4)	0.4121 (2)	0.0526 (9)	
H3	0.3323	0.0673	0.3931	0.063*	
C4	0.2152 (5)	0.1295 (4)	0.3523 (3)	0.0690 (11)	
H4A	0.1697	0.1757	0.3677	0.083*	
H4B	0.2069	0.0943	0.2932	0.083*	

### Atomic displacement parameters (Å^2^)

**Table:**

	*U*^11^	*U*^22^	*U*^33^	*U*^12^	*U*^13^	*U*^23^
Zn1	0.02154 (17)	0.02154 (17)	0.0372 (3)	0.01077 (8)	0.000	0.000
Br2	0.03343 (18)	0.03343 (18)	0.0332 (2)	0.01672 (9)	0.000	0.000
Br1	0.0489 (2)	0.0489 (2)	0.0351 (2)	0.02443 (11)	0.000	0.000
S1	0.0256 (4)	0.0211 (3)	0.0567 (4)	0.0089 (3)	0.0086 (3)	−0.0029 (3)
N2	0.0387 (14)	0.0236 (12)	0.0457 (11)	0.0164 (11)	0.0132 (11)	0.0082 (10)
N1	0.0420 (15)	0.0247 (12)	0.0473 (12)	0.0166 (12)	0.0169 (11)	0.0073 (10)
C2	0.0461 (19)	0.0288 (15)	0.0507 (15)	0.0236 (14)	0.0055 (14)	0.0045 (13)
C1	0.0317 (15)	0.0225 (14)	0.0312 (11)	0.0146 (12)	−0.0006 (11)	−0.0038 (10)
C3	0.061 (2)	0.0367 (18)	0.063 (2)	0.0268 (18)	0.0123 (18)	−0.0036 (16)
C4	0.079 (3)	0.058 (2)	0.059 (2)	0.026 (2)	−0.005 (2)	−0.0069 (18)

### Geometric parameters (Å, °)

**Table:**

Zn1—S1^i^	2.3426 (7)	N1—H1A	0.8600
Zn1—S1	2.3426 (7)	N1—H1B	0.8600
Zn1—S1^ii^	2.3426 (7)	C2—C3	1.499 (5)
Zn1—Br1	2.4640 (6)	C2—H2A	0.9700
S1—C1	1.727 (3)	C2—H2B	0.9700
N2—C1	1.318 (4)	C3—C4	1.278 (6)
N2—C2	1.452 (4)	C3—H3	0.9300
N2—H2	0.8600	C4—H4A	0.9300
N1—C1	1.327 (3)	C4—H4B	0.9300
			
S1^i^—Zn1—S1	116.038 (14)	N2—C2—H2A	108.2
S1^i^—Zn1—S1^ii^	116.038 (14)	C3—C2—H2A	108.2
S1—Zn1—S1^ii^	116.038 (13)	N2—C2—H2B	108.2
S1^i^—Zn1—Br1	101.64 (2)	C3—C2—H2B	108.2
S1—Zn1—Br1	101.64 (2)	H2A—C2—H2B	107.4
S1^ii^—Zn1—Br1	101.64 (2)	N2—C1—N1	120.5 (3)
C1—S1—Zn1	110.33 (10)	N2—C1—S1	116.9 (2)
C1—N2—C2	126.6 (3)	N1—C1—S1	122.6 (2)
C1—N2—H2	116.7	C4—C3—C2	127.0 (4)
C2—N2—H2	116.7	C4—C3—H3	116.5
C1—N1—H1A	120.0	C2—C3—H3	116.5
C1—N1—H1B	120.0	C3—C4—H4A	120.0
H1A—N1—H1B	120.0	C3—C4—H4B	120.0
N2—C2—C3	116.3 (3)	H4A—C4—H4B	120.0
			
S1^i^—Zn1—S1—C1	141.40 (9)	C2—N2—C1—S1	−179.4 (2)
S1^ii^—Zn1—S1—C1	−0.08 (10)	Zn1—S1—C1—N2	145.88 (19)
Br1—Zn1—S1—C1	−109.34 (9)	Zn1—S1—C1—N1	−35.4 (2)
C1—N2—C2—C3	−75.8 (4)	N2—C2—C3—C4	−1.8 (6)
C2—N2—C1—N1	1.8 (4)		

Symmetry codes: (i) −*x*+*y*, −*x*+1, *z*; (ii) −*y*+1, *x*−*y*+1, *z*.
